# Porcine Reproductive and Respiratory Syndrome in Hybrid Wild Boars, China

**DOI:** 10.3201/eid1706.101518

**Published:** 2011-06

**Authors:** Jiaqiang Wu, Shaoning Liu, Shun Zhou, Zhao Wang, Kun Li, Yuyu Zhang, Jiang Yu, Xiaoyan Cong, Xiaowei Chi, Jun Li, Shaojian Xu, Yijun Du, Sufang Ren, Jinbao Wang

**Affiliations:** Author affiliations: Shandong Academy of Agricultural Sciences, Jinan, People’s Republic of China (J. Wu, Y. Zhang, X. Cong, J. Li, S. Xu, Y. Du, S. Ren, J. Wang);; Shandong Key Laboratory of Animal Disease Control and Breeding, Jinan (J. Wu, S. Liu, K. Li, Y. Zhang, J. Yu, X. Cong, J. Li, Y. Du, S. Ren, J. Wang);; Shandong Agricultural University, Tai’an, People’s Republic of China (S. Liu, J. Wang);; Qingdao Agricultural University, Qingdao, People’s Republic of China (S. Zhou, Z. Wang, K. Li, J. Yu);; Shandong University of Traditional Chinese Medicine, Jinan (X. Chi)

**Keywords:** porcine reproductive and respiratory syndrome, porcine reproductive and respiratory syndrome virus, viruses, hybrid wild boars, epidemiology, experimental infection, China, dispatch

## Abstract

We conducted a serologic investigation of porcine reproductive and respiratory syndrome virus (PRRSV) in hybrid wild boar herds in China during 2008–2009. PRRSV isolates with novel genetic markers were recovered. Experimental infection of pigs indicated that hybrid wild boars are involved in the epidemiology of PRRSV.

Hybrid wild boars, also known as special wild pigs in China, are a hybrid animal with 75% wild boar lineage and 25% domestic Duroc pig lineage. Hybrid wild boars are genetically stable and retain the appearance of wild boars. In addition, they have less body fat, are strongly adaptable to various environments, and have an estrus cycle in all 4 seasons.

We observed an increased incidence of high fever and respiratory disorders in hybrid wild boars. This illness resulted in increased economic losses in Shandong Province, China. We conducted a serologic study and identified highly pathogenic porcine reproductive and respiratory syndrome virus (PRRSV) in affected hybrid wild boars.

## The Study

During April 2008–December 2009 in Shandong Province, 613 blood samples were obtained from hybrid wild boars that had not been vaccinated against PRRSV (Table). The Herd Check PRRS commercial ELISA kit (IDEXX Laboratories, Norcross, GA, USA) was used for serologic analysis; 167 (27.2%) of 613 samples were seropositive for PRRSV, and 2 PRRSV isolates (TAYZ and ZCYZ) were obtained. The viruses were plaque-purified once in MARC-145 cells and amplified for sequencing and infection of other pigs.

**Table:** Serologic prevalence of PRRSV in hybrid wild boars, China, April 2008–December 2009*

No. pigs/ herd	Farm environment	No. farms tested	No. positive samples/ no. tested (%)
70–99	Hill	4	19/81 (23.5)
	Slope	6	33/122 (27.0)
	Forest	3	17/60 (28.3)
100	Hill	6	37/135 (27.4)
	Slope	4	29/97 (29.9)
	Forest	5	32/118 (27.1)
Total		28	167/613 (27.2)

*PRRSV, porcine reproductive and respiratory syndrome virus.

Because open reading frame 5 (ORF5) and the nonstructural protein 2 (NSP2) gene are 2 of the most variable genes in PRRSV (*1**–**3*), these genes were sequenced to identify genetic variation in the 2 isolates. Primers and reverse transcription PCR were used as described (*4*). Sequences for NSP2 and ORF5 genes were submitted to GenBank (accession nos. HM854221 and HM854224 for TAYZ and HQ388394 and HQ384170 for ZCYZ). Nucleotide sequences were analyzed by using MEGA 4.0 (www.megasoftware.net).

**Figure 1:**
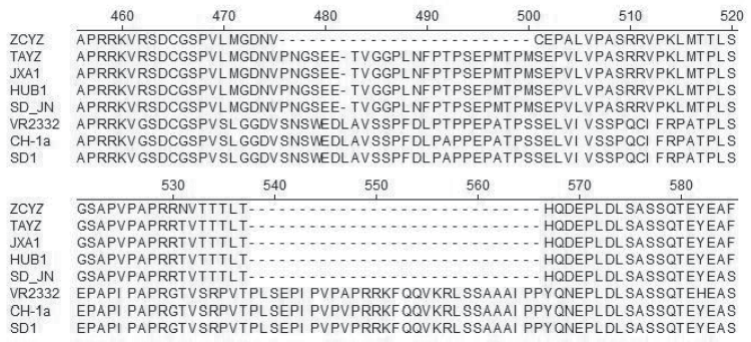
Amino acid sequence alignments of partial nonstructural protein 2 genes of porcine reproductive and respiratory syndrome virus isolates, China. Dashes indicate deletions.

TAYZ and ZCYZ had higher nucleotide homologies in ORF5 with highly pathogenic PRRSV (prototype virus JXA1) than North American-type PRRSV (prototype virus VR2332), and European-type PRRSV (prototype virus LV). For NSP2 of TAYZ, 2 deletions were identified: a 1-aa deletion at position 482, and a 29-aa deletion at positions 533–561. These deletions were similar to those in JXA1, HUB1, and SD-JN isolates associated with porcine high fever disease reported in China (*4**–**7*). For NSP2 of ZCYZ, 2 deletions were identified: a novel 25-aa deletion at positions 476–500, and a 29-aa deletion at positions 533–561 (Figure 1).

To determine the virulence and pathogenesis of the PRRSV isolated, we performed experimental infection with the ZCYZ isolate in ten 6-week-old domestic Duroc crossbred pigs and ten 6-week-old hybrid wild boars. The animal infection protocol was reviewed and approved by the Shandong Province Animal Ethics Committee. Domestic pigs and hybrid wild boars were randomly divided into 2 groups, each consisting of 5 pigs. These pigs were shown by serologic analysis to be negative for antibodies against PRRSV, swine influenza virus, and mycoplasmas.

In hybrid wild boars injected intramuscularly with 1 mL of ZCYZ isolate (10^3^ 50% tissue culture infectious doses/mL), high fever >40.8°C and respiratory disorders were observed at 5–6 days postinfection (dpi). Two boars died at 8 dpi and 1 boar died at 9 dpi. Two pigs were killed at 21 dpi and autopsies were then performed.

**Figure 2:**
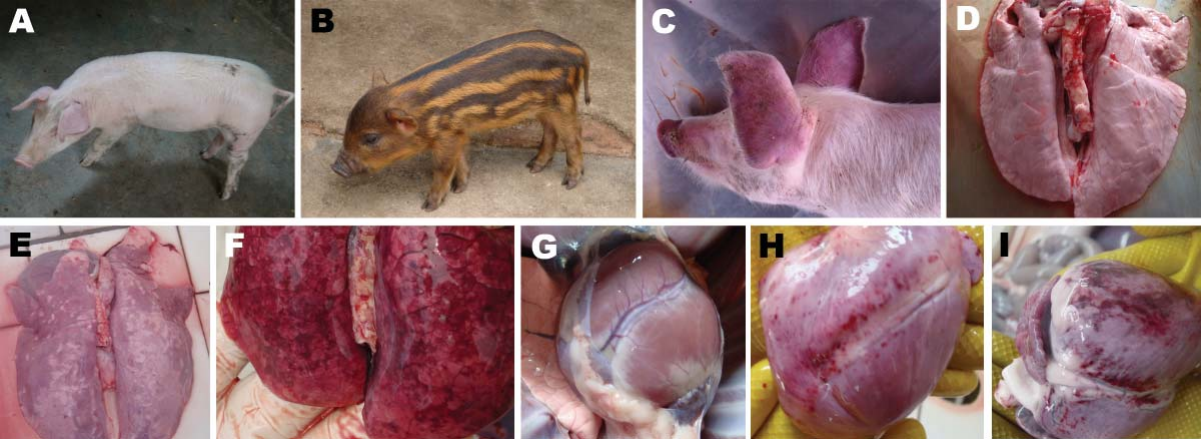
Experimental infection of domestic Duroc crossbred pigs and hybrid wild boars with porcine reproductive and respiratory syndrome virus isolate ZCYZ. A) Domestic Duroc crossbred pig (control) injected with Dulbecco minimal essential medium (DMEM), showing normal skin color. B) Hybrid wild boar (control) injected with DMEM, showing normal skin color. C) Red discoloration in ears of a domestic Duroc crossbred pig infected with ZCYZ). D) Normal pathologic appearance of lungs in a control domestic Duroc crossbred pig. E) Pulmonary hyperplasia and consolidation in a hybrid wild boar infected with ZCYZ. F) Edema and hemorrhage in the lungs of domestic Duroc crossbred pig infected with ZCYZ). G) Normal pathologic appearance of heart in a control hybrid wild boar. H) Cardiac hemorrhage and edema in a hybrid wild boar infected with ZCYZ. I) Hemorrhagic spots in heart of domestic Duroc crossbred pig infected with ZCYZ.

Pulmonary hyperplasia and consolidation (Figure 2, panel E) and cardiac hemorrhage and edema (Figure 2, panel H) were observed in the dead hybrid wild boars. PRRSV-specific antibodies were detectable by 7 dpi, had increased by 14 dpi, and remained high until autopsy at 21 dpi. For the infected domestic Duroc crossbred pigs, high fever >41°C and respiratory problems were observed at 4–5 dpi, and red discoloration was observed in the ears (Figure 2, panel C). Two pigs in this group died at 6 dpi; the other 3 pigs in this group died at 7 dpi. The dead pigs all had multiple lesions in various organs, such as edema and hemorrhage in the lung (Figure 2, panel F) and hemorrhagic spots in the heart (Figure 2, panel I). Duroc crossbred pigs and hybrid wild boars in the control group injected with Dulbecco minimal essential medium had a normal appearance (Figure 2, panels A, B), appetite, and rectal temperature during the experiment, and no obvious pathologic changes were observed in the lungs (Figure 2, panel D) and heart (Figure 2, panel G).

## Conclusions

PRRSV was identified in hybrid wild boars during our serologic study. Prevalence of antibodies against PRRSV was similar for different types of farm environments (Table). Two PRRSV isolates were obtained from hybrid wild boars; 1 of the isolates (ZYCZ) has a novel genetic marker in the NSP2 gene. The ZCYZ isolate showed high virulence in domestic Duroc crossbred pigs and hybrid wild boars. The virulence of the TAYZ isolate has not yet been determined.

Shandong Province is one of the major pig-producing areas in China; annual production is 60 million domestic pigs and 500,000 hybrid wild boars. It has been reported that 793 (59.5%) of 1,332 serum samples from domestic pigs were seropositive for PRRSV, and seropositivity was >80% in some large-scale commercial production farms in the Shandong area (*8*). As a transitional group between domestic pigs and wild boars, hybrid wild boars were sensitive to highly pathogenic PRRSV. However, 167 (only 27.2%) of 613 samples from hybrid wild boars were positive for PRRSV, indicating that the prevalence of PRRSV differed between domestic pigs and hybrid wild boars in this area. Hybrid wild boars are usually raised on hills or slopes or in forests, characterized by a relatively low-density pig population and separation from commercial domestic pigs. These features may have helped to limit transmission of PRRSV to hybrid wild boar herds.

When PRRSV enters the wild pig population, its subsequent spread has been found to be limited, probably because the virus is not easily transmitted within a low-density or medium-density population (*9*). Some genetic traits in local wild boars may have been inherited over decades or even for hundreds of years. Whether these traits confer resistance to PRRSV remains unknown and warrants further study.

PRRSV is currently divided into 2 distinct genotypes, European and North American, which show ≈60% nt sequence similarity (*10*). In this study, the 2 PRRSV isolates from hybrid wild boars belonged to the North American genotype on the basis of sequence analysis and were similar to highly pathogenic PRRSV isolated during 2006–2009 in China. This finding suggests that infection of hybrid wild boars with PRRSV may be caused by the spread of highly pathogenic PRRSV in domestic pigs in recent years.

Experimental infection of pigs showed that the ZCYZ isolate was highly pathogenic in domestic pigs and hybrid wild boars, indicating that hybrid wild boars likely play a role in the epidemiology of PRRSV and may be an alternative model that can be used to study transmission of PRRSV among the wild boar population. However, how PRRSV has evolved and varies in hybrid wild boars is not clear, and further studies, including extensive genomic sequence analyses, should be conducted.
